# Antimicrobial and antioxidant properties of *Boswellia sacra* resin: extraction, evaluation, and formulation into a topical cream for dermatological applications

**DOI:** 10.3389/fmed.2025.1664265

**Published:** 2025-08-25

**Authors:** Abdulhakim Al-Saban, Abdullah H. Maad, Ali Salman Al-Sham, Hammood Mohamed Saad

**Affiliations:** ^1^Department of Pharmacy, Jiblah University for Medical and Health Science, Ibb, Yemen; ^2^Department of Pharmaceutics, College of Pharmacy, University of Al-Ameed, Karbala, Iraq; ^3^Department of Pharmacology, Faculty of Medicine, Sanaa University, Sanaa, Yemen

**Keywords:** *frankincense* resin, antibacterial activity, cream formulation, anti-oxidant, medicinal plants

## Abstract

**Background:**

*Frankincense* (*Boswellia sacra*) Resin has been used in traditional medicine for millennia because of its anti-inflammatory, antibacterial, and wound-healing characteristics. Recent research has proved its medicinal promise, particularly against resistant bacterial strains and oxidative stress.

**Objective:**

This study seeks to assess the antimicrobial and antioxidant properties of *Boswellia sacra* resin, extracted with ethanol, and to formulate a topical cream for dermatological use, specifically targeting skin infections and inflammatory conditions such as acne.

**Methods:**

Two techniques were used to extract *frankincense* resin: maceration and Soxhlet. The well diffusion method was used to evaluate the antibacterial activity against *Escherichia coli, Bacillus cereus, Pseudomonas aeruginosa, and Staphylococcus aureus.* The DPPH radical scavenging test was used to determine antioxidant activity. We produced and assessed the physical characteristics of a cream formulation containing *Boswellia sacra* extract, such as texture, homogeneity, and spreadability.

**Results:**

The ethanol extract of *Boswellia sacra* demonstrated notable antibacterial efficacy, especially against *Staphylococcus aureus*, with an inhibition zone measuring 15 mm at a dosage of 100 mg/mL. The antioxidant activity exhibited a dose-dependent relationship, with Soxhlet extraction demonstrating superior radical scavenging activity (84.66%) relative to maceration. The developed cream exhibited superior organoleptic characteristics, such as homogeneity, smoothness, and spreadability, suggesting its suitability for topical use.

**Conclusion:**

The results validate the notable antibacterial and antioxidant characteristics of *Boswellia sacra* resin. The formulated cream is a viable option for addressing skin infections and inflammatory illnesses, integrating traditional applications with contemporary pharmacological compositions.

## Introduction

Historically, ethnomedicine has made extensive use of the plant’s whole spectrum of parts, including its bark, fruit, leaves, stem, and roots, all of which have therapeutic qualities. People recognise the use of herbal medicine and botanical extracts as a substitute for synthetic or pharmaceutical medications, often due to their reduced side effects. The evidence indicates that the application of herbal medicine techniques aligns with a resurgence in natural remedies that typically have fewer or no side effects. It is extensively described in the Vedic literature for the treatment of various diseases (1, 2). *Frankincense,* which is also referred to as olibanum, is a substance that is extracted from the bark of trees in the Boswellia genus, with a particular emphasis on *Boswellia sacra* and *Boswellia serrata*. *Frankincense* has been a critical element in traditional medicine and various cultural practices for more than 5,000 years. It is highly regarded for its therapeutic properties, which include anti-inflammatory, antimicrobial, and antioxidant effects. It has been employed to treat various conditions, including arthritis, asthma, skin infections, and even cancer prevention, in regions such as India, Africa, and the Middle East (3, 4). It is extensively described in the Vedic literature for the treatment of various diseases (5). Recent research employing rigorous scientific methodologies has substantiated these conventional applications. For example, Al-Harrasi et al. (3) emphasised the broad-spectrum antimicrobial activity of *Boswellia sacra*, which is notably effective against drug-resistant bacteria, such as MRSA. In the same vein, Di Stefano et al. (6) demonstrated that *frankincense* oils sourced from various locations possessed substantial antibacterial properties. Their research indicated that the chemical composition of *frankincense* oils is contingent upon their geographic origin, which may affect their efficacy against various pathogens. In addition, (7) demonstrated that the resins from *Boswellia* species possess substantial antibacterial and antifungal properties, particularly when extracted with ethanol. *Frankincense has* demonstrated promising antioxidant activity in addition to its antimicrobial properties. Shen et al. (4) verified that *frankincense* extracts, particularly those that are high in phenolic compounds, possess robust free radical scavenging capabilities. These properties are responsible for their protective effects against oxidative stress and cellular injury. These results are consistent with previous research that has suggested the presence of antioxidants, including flavonoids and boswellic acids, which are recognised for their protective functions in skin health and anti-inflammatory properties (12). The increasing body of evidence emphasises the potential of *frankincense as* a medicinal agent and as a constituent in skin care formulations, thereby increasing the development of natural therapeutic products. The incorporation of *frankincense* extracts into topical formulations, particularly for conditions such as acne, dermatitis, and other inflammatory skin disorders, has been driven by the growing demand for natural cosmetics. Zhang et al. (8) emphasised the efficacy of *frankincense-based* moisturisers in treating acne, citing their capacity to promote wound healing and reduce inflammation. The objective of the development of these formulations is to leverage the dual function of *frankincense* as a cosmetic and therapeutic ingredient, providing a natural alternative to synthetic treatments. In addition, the demand for natural skincare formulations is increasing, and plant extracts that offer both cosmetic and therapeutic benefits are being incorporated into topical applications. *Frankincense,* a crucial ingredient in contemporary herbal skincare preparations, is recognised for its bioactive composition and aromatic profile. According to Rahman et al. (9), moisturisers that contain *Boswellia* extracts enhance wound healing and reduce inflammation, which is consistent with prior ethnobotanical observations. The use of phytocosmetics that capitalise on the antioxidant and antimicrobial properties of these substances is on the rise, offering safer and more effective alternatives to synthetic products (10). This investigation expands upon these discoveries by investigating the extraction of *Boswellia sacra* resin, assessing its antioxidant and antimicrobial properties, and transforming it into a product for dermatological use. The objective is to offer a contemporary, scientifically supported method of using *frankincense* in the treatment of skin infections and inflammatory conditions, in accordance with the most recent trends in natural medicine and cosmetics.

## Methodology

### Preparation of the sample

#### Sample preparation

The *frankincense* resin utilised in this study is derived from *Boswellia sacra*, a species indigenous to arid, mountainous areas of Yemen. The resin was obtained from local markets in Sana’a, Yemen, where *Boswellia sacra* is typically harvested. The resin was identified and authenticated according to its morphological characteristics, which align with descriptions in the literature for *Boswellia sacra* (3). The resin samples were acquired from local vendors with a longstanding history of harvesting and trading *frankincense* in the region. The species was verified via visual inspection and local expertise regarding the plant. After collection, the resin underwent a cleaning process to eliminate dirt and extraneous materials, followed by shade-drying to maintain its bioactive compounds. The dried resin was crushed into a coarse powder with a mortar and pestle, preparing the material for the extraction process.

### Extraction of resin *frankincense*

The ethanol extraction was performed using two different techniques: maceration and Soxhlet extraction, to compare the efficiency of each method in extracting the bioactive compounds from the *frankincense* resin.

#### Maceration method

- The dried *frankincense* resin (200 g) was finely powdered and mixed with 300 mL of 96% ethanol in a glass container.
- The mixture was allowed to stand at room temperature for 72 h, with intermittent shaking every 12 h to ensure efficient extraction of the resin’s active compounds.
- After the extraction period, the solution was filtered through Whatman No. 1 filter paper to remove any solid debris, and the resulting ethanol extract was collected and stored for further use.

#### Soxhlet extraction method

- In this method, 50 g of powdered *frankincense* resin was placed in the extraction chamber of the Soxhlet apparatus, and 300 mL of 96% ethanol was added to the solvent flask.
- The extraction was carried out at a temperature of approximately 80 °C, with solvent cycling for 6 h. This method ensures a more thorough extraction of the active compounds from the resin.
- After the completion of the extraction, the ethanol extract was collected and filtered to remove any solid particles, and the resulting solution was stored at room temperature for subsequent analysis.

### Phytochemical screening

We conducted phytochemical screening to determine the presence of several bioactive components in the *frankincense* resin. We used the following methods to screen the extracts:

1. Test for steroids: We used the Salkowski and Liebermann-Burchard tests to check for the presence of steroids in the extracts. When a few drops of strong sulphuric acid were applied to the extract in the Salkowski test, the presence of steroids was determined by the production of a reddish-purple colour. In the Liebermann-Burchard test, the extract was heated after acetic anhydride and sulphuric acid were added; the presence of steroids was confirmed by a change in colour to either green or blue.
2. Test for triterpenoids: Triterpenoids were detected using the Salkowski and Liebermann-Burchard tests, similar to steroids, where a color change indicated their presence.
3. Test for saponins: Saponins were detected using the foam test. A minimal quantity of the extract was agitated with water, and the development of stable foam signified the presence of saponins.
4. Test for alkaloids: Alkaloids were evaluated via Dragendorff’s reagent and Mayer’s reagent. The production of an orange precipitate with Dragendorff’s reagent and a white precipitate with Mayer’s reagent showed a favourable reaction.
5. Test for flavonoids: Flavonoids were identified using the alkaline reagent test. A yellow coloration that turns colorless upon acidification indicated the presence of flavonoids.
6. Test for tannins: Tannins were detected by adding a few drops of 10% lead acetate solution to the extract. The formation of a white precipitate confirmed the presence of tannins.
7. Test for glycosides: The Keller-Killiani test was employed to identify glycosides. A reddish-brown hue at the interface of the extract and sulphuric acid signified the presence of glycosides.
8. Test for carbohydrates: The presence of carbohydrates was confirmed using Fehling’s solution, which produced a brick-red precipitate in the presence of reducing sugars.
9. Test for proteins and amino acids: The Biuret test, which turns violet or purple when peptide bonds are present, was used to identify proteins and amino acids.

### Antimicrobial activity testing

#### Preparation of microbial suspensions

Microbial strains were incubated in nutritional broth (for bacteria) and Sabouraud dextrose broth (for fungus) overnight at 37 °C. The microbial suspensions were calibrated to a concentration of about 1 × 10^8^ CFU/mL using a spectrophotometer, guaranteeing consistent bacterial and fungal concentrations across all assays.

#### Agar well diffusion method

1. Preparation of agar plates: Muller-Hinton agar (MHA) plates were prepared and inoculated with 100 μL of the standardized microbial suspension, spread evenly using a sterile glass rod.
2. Preparation of extracts: The ethanol extracts derived from the maceration and Soxhlet methods were diluted to four concentrations: 100, 75, 50, and 25 mg/mL, using dimethyl sulfoxide (DMSO) as the solvent. A ciprofloxacin disc (10 μg) served as the positive control, whereas DMSO functioned as the negative control.
3. Inoculation of plates: Employing a sterile cork borer, wells measuring 6 mm in diameter were created on the agar plates, subsequently filled with 100 μL of each extract at varying concentrations.
4. Incubation: The inoculated dishes were incubated at 37 °C for 24 h (bacteria) and 48 h (fungi).
5. Evaluation of results: After incubation, the diameter of the inhibition zone around each well was measured in millimeters. The inhibition zone was indicative of the antimicrobial efficacy of the extract. The larger the inhibition zone, the greater the antimicrobial activity.

### Antioxidant activity testing

The antioxidant capacity of ethanol extracts obtained using maceration and Soxhlet techniques was assessed using the DPPH (2,2-diphenyl-1-picrylhydrazyl) radical scavenging test, a standard technique for evaluating the antioxidant potential of plant extracts.

#### DPPH radical scavenging assay

1. Preparation of DPPH solution: A 90 μM solution of DPPH in methanol was prepared. The DPPH radical is a stable free radical that shows strong absorbance at 517 nm.
2. Preparation of extracts: The ethanol extract of *frankincense* resin was diluted to final concentrations of 100, 75, 50, 25, 12.5, and 6.25 mg/mL using methanol.
3. Reaction: 1 mL of each sample was combined with 1 mL of DPPH solution, and the reaction mixture was incubated at ambient temperature for 30 min in the absence of light.
4. Measurement of absorbance: The absorbance of the reaction mixture was measured at 517 nm using a spectrophotometer. Ascorbic acid (vitamin C) was used as a positive control.
5. Calculation of inhibition percentage: The antioxidant activity was expressed as the percentage of DPPH radical inhibition, calculated using the following formula: $I\%=(\frac{A^{{}^{\circ}}-A}{A^{{}^{\circ}}})\times 100.$ Where A^∘^ is the absorbance of the DPPH control, and A is the absorbance of the sample solution.
6. IC50 determination: The IC50 value, which is the concentration required to scavenge 50% of the DPPH radicals, was determined by plotting inhibition percentage against concentration.

#### Formulation of *frankincense* resin cream

To explore the practical application of the *Boswellia sacra* resin as a topical product, a cream formulation was developed incorporating the ethanol extract.

##### Ingredients used

Oil phase:

- Shea butter: 45 g
- Coconut oil: 2.5 g
- Jojoba oil: 2.5 g
- Acetyl alcohol: 5 g
- *Boswellia sacra* extract (from Soxhlet extraction): 2 g

Water phase:

- Cetrimide: 1 g
- Distilled water: 45 g

### Cream preparation process

1. Preparation of the oil phase: Shea butter (45 g) was heated in a water bath at 70–75 °C until it melted. The remaining oils—coconut oil (2.5 g), jojoba oil (2.5 g), and acetyl alcohol (5 g)—were added to the melted shea butter and mixed until homogeneous.
2. Preparation of the water phase: Cetrimide (1 g) was dissolved in distilled water (45 g) and heated to the same temperature (70–75 °C).
3. Emulsification: The oil phase was added gradually to the water phase while stirring continuously with a glass rod. The mixture was stirred until it cooled and solidified, resulting in a smooth and homogeneous cream.
4. Evaluation of physical properties:

◦ Color: The color of the cream was observed visually (white, with a slight yellowish tint from the *frankincense* extract).
◦ Odor: The aroma of the cream was evaluated for its aromatic characteristics.
◦ Texture: The texture of the cream was evaluated by touch, ensuring that it was smooth and spreadable.
◦ Homogeneity: The cream was assessed for uniformity, ensuring no separation of phases.
◦ Spreadability: The ability of the cream to spread evenly on the skin was tested by applying a small amount to the forearm.

### Data analysis

All experiments were conducted in triplicate. The data were presented as the mean ± standard deviation (SD). Statistical analysis was conducted using one-way ANOVA, followed by post-hoc Tukey’s test to evaluate multiple comparisons between treatment groups. Statistical significance was defined as a *p*-value of less than 0.05.

## Results

Table 1 illustrates that both the maceration and Soxhlet extraction techniques yielded extracts with a diverse array of bioactive components, such as sterols, triterpenoids, flavonoids, tannins, and alkaloids. The existence of these phytochemicals supports the antibacterial, antioxidant, and anti-inflammatory properties of the resin, which are crucial for its medicinal efficacy.

**Table 1:** Phytochemical screening of *Boswellia sacra.*

Phytochemical constituent	Test reagent	Crude extracts	
		Ethanol by mace.	Ethanol by sox.
Steroids	Salkowski	+	+
	Lieberman’s	+	+
Triterpenoids	Salkowski	+	+
	Lieberman’s	+	+
Saponin	Foam test	+	+
Alkaloid	Dragendroffs	+	+
	Mayer’s	+	+
Flavonoid	Alkaline	+	+
Tannins	Lead acetate 10%	+	+
Glycosides	Keller-kiliani	+	+
Carbohydrates	General	+	+
	Fehling	+	+
Proteins and amino acids	Biuret	+	+

(+) presence, (−) not presence, (mace): maceration, (sox): Soxhlet.

Table 2 demonstrates that the maceration extract displayed significant antibacterial activity against *Staphylococcus aureus*, with a 15 mm inhibition zone at a dosage of 100 mg/mL, indicating strong antimicrobial characteristics. Other bacterial strains, such as *Pseudomonas aeruginosa*, *Bacillus cereus*, and *Escherichia coli*, exhibited modest antimicrobial activity, with inhibition zones between 9 mm and 14 mm at elevated concentrations (100 mg/mL). The lack of antifungal action against *Candida albicans* suggests that *frankincense* extracts may be more effective against bacterial infections than fungal infections.

**Table 2:** Antimicrobial efficacy of *Boswellia sacra* resin extracts (maceration method) against selected bacterial and fungal strains.

Concentration (mg/mL)	*Staphylococcus aureus*	*Pseudomonas aeruginosa*	*Bacillus cereus*	*Escherichia coli*	*Candida albicans*
100	15 mm	14 mm	13 mm	12 mm	No Activity
75	14 mm	13 mm	12 mm	11 mm	No Activity
50	12 mm	11 mm	11 mm	9 mm	No Activity
25	10 mm	9 mm	8 mm	7 mm	No Activity

Table 3 indicates that the Soxhlet extract demonstrated significant antibacterial activity, with inhibition zones of 15 mm for *Staphylococcus aureus* and 16 mm for *Bacillus cereus* at a concentration of 100 mg/mL, signifying greater efficacy relative to the maceration approach. The inhibition zones for *Pseudomonas aeruginosa* and *Escherichia coli* were significant, measuring 14 mm and 15 mm, respectively, indicating that Soxhlet extraction is very efficacious against both gram-positive and gram-negative bacteria. Like the maceration extract, the Soxhlet extract had negligible antifungal activity against *Candida albicans*.

**Table 3:** Antimicrobial efficacy of *Boswellia sacra* resin extracts (Soxhlet method) against selected bacterial and fungal strains.

Concentration (mg/mL)	*Staphylococcus aureus*	*Pseudomonas aeruginosa*	*Bacillus cereus*	*Escherichia coli*	*Candida albicans*
100	15 mm	14 mm	16 mm	15 mm	No Activity
75	14 mm	13 mm	14 mm	13 mm	No Activity
50	13 mm	12 mm	13 mm	12 mm	No Activity
25	11 mm	9 mm	12 mm	10 mm	No Activity

Table 4 illustrates that the Soxhlet extract displayed markedly greater antioxidant activity than the maceration extract, with a radical scavenging activity (RSA) of 84.66% at 100 mg/mL, highlighting its enhanced ability to neutralise free radicals. The maceration extract exhibited a radical scavenging activity (RSA) of merely 28.43% at 100 mg/mL, signifying inferior antioxidant efficacy and implying that Soxhlet extraction is more effective in isolating antioxidant chemicals from the resin. Ascorbic acid (Vitamin C), employed as a positive control, exhibited the highest antioxidant activity (90.09% RSA), whereas the Soxhlet extract demonstrated similar efficacy, illustrating the importance of *frankincense* as a natural antioxidant.

**Table 4:** Antioxidant activity of *Boswellia sacra* extracts using DPPH assay.

Concentration (mg/mL)	Soxhlet extract RSA (%)	Maceration extract RSA (%)	Ascorbic acid RSA (%)
100	84.66%	28.43%	90.09%
75	68.47%	23.74%	63.89%
50	48.88%	21.61%	48.88%
25	42.17%	19.38%	42.17%
12.5	18.53%	7.34%	18.53%
6.25	11.39%	4.15%	11.39%

Table 5 demonstrates that the Soxhlet extract displayed a lower IC50 value (45 mg/mL) than the maceration extract (120 mg/mL), indicating superior antioxidant capabilities. This finding corroborates the concept that Soxhlet extraction is superior in isolating phenolic and antioxidant-rich chemicals from the resin.

**Table 5:** IC50 values for DPPH radical scavenging activity.

Extract type	IC50 value (mg/mL)
Soxhlet extract	45 mg/mL
Maceration extract	120 mg/mL

**Figure 1:**
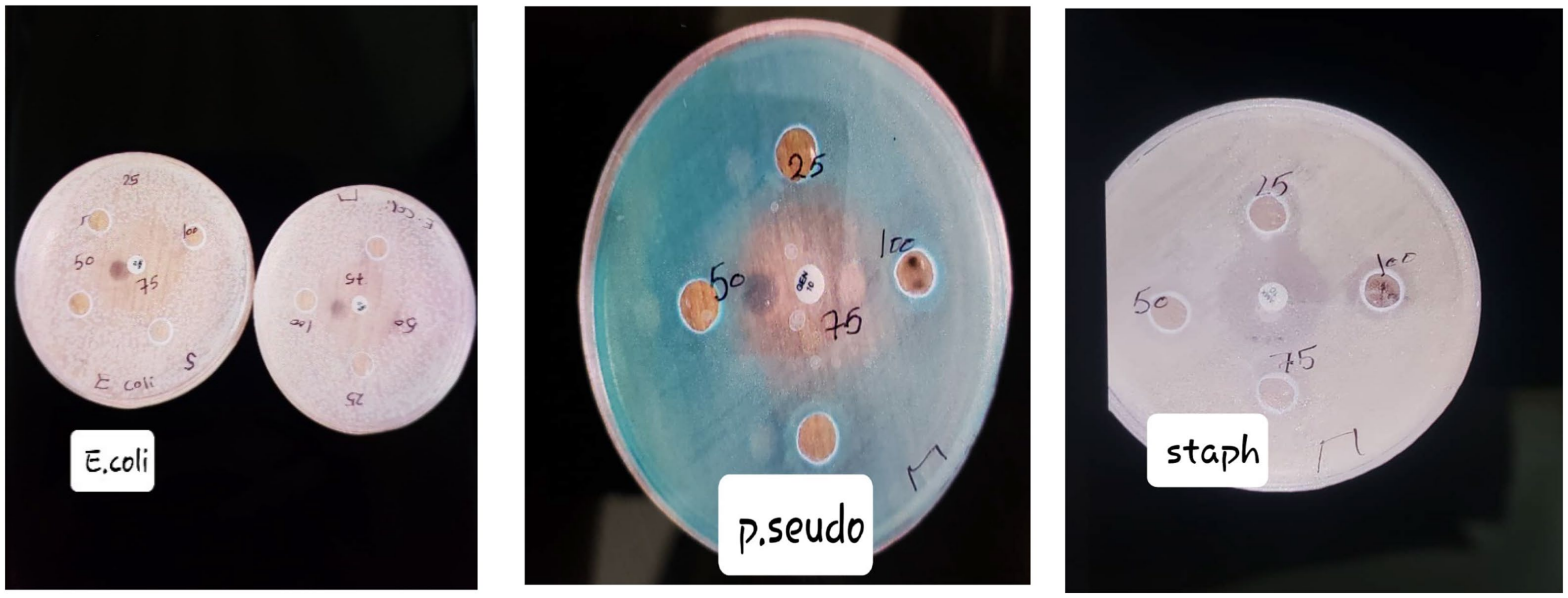
Inhibition zones around wells containing the *frankincense* extract at various concentrations for each microorganism.

Figure 1 shows the *frankincense* extract has antibacterial characteristics, which become more effective as concentrations increase. The extract appears to be more effective against some microbes, indicating its potential as a selective antimicrobial agent.

Table 6 demonstrates that *Boswellia sacra* extract possesses notable antioxidant capabilities, with elevated concentrations leading to enhanced radical scavenging activity. The ascorbic acid approach had the most antioxidant potential, succeeded by the Soxhlet and maceration procedures for efficacy.

**Table 6:** Radical scavenging activity of *Boswellia sacra* extract.

*Boswellia sacra* extract concentrati on (mg/mL)	Absorbance at 517 nm (maceration)	Radical scavenging activity% (maceration)	Absorbance at 517 nm (Soxhlet)	Radical scavenging activity % (Soxhlet)	Absorbance at 517 nm (ascorbic acid)	Radical scavenging activity % (ascorbic acid)
100	0.672	28.43	0.144	84.66	–	–
75	0.716	23.74	0.296	68.47	–	–
50	0.726	22.68	0.339	63.89	0.093	90.09
25	0.737	21.61	0.480	48.88	–	–
12.5	0.757	19.38	0.543	42.17	–	–
6.25	0.870	7.34	0.765	18.53	–	–
3.12	0.900	4.15	0.832	11.39	–	–

Table 7 illustrates that the cream formulation with *Boswellia sacra* extract had advantageous physical characteristics, such as a smooth texture, consistent uniformity, and a non-greasy composition, rendering it appropriate for cosmetic uses. The cream displayed the characteristic fragrant fragrance of *frankincense,* augmenting its attractiveness in cosmetic compositions. Moreover, the cream exhibited superior spreadability, an essential attribute for efficient topical application, indicating its potential utility in dermatological treatments such as acne or mild infections.

**Table 7:** Physical characteristics of *Boswellia sacra* extract-based cream formulation.

Parameter	Cream
Color	White
Odor	Aromatic
Texture	Soft
Homogeneity	Excellent
Consistency	Excellent
Spread ability	Good/Excellent

## Discussion

Herbal medicine has contributed numerous powerful medications to the extensive drug arsenal of contemporary medical research worldwide. Both in crude form and as a pure chemical on which modern medicines are structured (11). According to the results of the antimicrobial tests, *Boswellia sacra* maceration and Soxhlet extracts demonstrated strong antibacterial activity, especially against two important skin pathogens that are known to cause infections like cellulitis, acne, and abscesses *Staphylococcus aureus* and *Pseudomonas aeruginosa*. Both extraction techniques generated 15 mm inhibition zones against *Staphylococcus aureus* at the maximum dose (100 mg/mL), suggesting potent antibacterial qualities. However, the Soxhlet extract showed marginally greater efficacy, with inhibitory zones for *Bacillus cereus* reaching up to 16 mm. This further supports the Soxhlet extraction method’s superior extraction efficiency for bioactive chemicals. Furthermore, the study revealed minimal antifungal efficacy against the common fungus strain *Candida albicans*, which is associated with skin infections. This result is in line with past research that indicates Boswellia extracts typically have more potent antibacterial than antifungal effects (4, 7). This finding could be because *frankincense* contains particular bioactive components, like boswellic acids and triterpenoids, that work better against bacterial cell wall structures than fungal cell membranes.

The antioxidant capacity of *Boswellia sacra* was determined using the DPPH radical scavenging assay, which assessed the extract’s ability to neutralise free radicals. At 100 mg/mL, the Soxhlet extract had 84.66% radical scavenging activity, which was much higher than the maceration extract (28.43%). This result shows that the Soxhlet approach, which employs continuous solvent cycling, is more effective at extracting active antioxidant chemicals, most likely phenolic compounds and flavonoids, from the resin. The Soxhlet extract’s increased antioxidant activity demonstrates the resin’s potential as a natural antioxidant source, which can help protect the skin from oxidative stress and free radical ageing.

The Soxhlet extract had an IC50 value of 45 mg/mL, demonstrating that it is effective at scavenging free radicals even at low concentrations, making it a promising ingredient for antioxidant cosmetic compositions. When compared to ascorbic acid (90.09% RSA), *frankincense* extract is more potent, although ascorbic acid is still one of the most effective natural antioxidants, highlighting *frankincense*‘s usefulness as a competitive alternative.

The effective development of a topical cream with *Boswellia sacra* extract showcased its viability for practical dermatological application. The cream demonstrated superior texture, spreadability, and homogeneity, which are essential attributes for efficient topical applications. The fragrant scent of *frankincense* enhanced the product’s inherent perfume, a sought-after attribute in cosmetics. The non-greasy consistency and seamless application render it an optimal choice for addressing skin ailments such as acne, minor injuries, and inflammation.

The cream’s stability, as seen by the absence of phase separation, guarantees that the active ingredients are evenly dispersed, resulting in consistent therapeutic effects with each application. The cream’s antioxidant and antibacterial qualities, which are derived from *Boswellia sacra* extract, increase its potential as a natural skincare cure. The results of this study are consistent with the findings of numerous previous studies on *Boswellia* species, including the work of Al-Harrasi et al. (3), which reported significant antimicrobial activity against *Staphylococcus aureus*, and Shen et al. (4), which confirmed the antioxidant potential of *frankincense* extracts. In addition, Rahman et al. (9) successfully integrated *frankincense* extract into a topical salve for wound healing, thereby substantiating the notion that *Boswellia sacra* can be effectively included in dermatological formulations.

This study extends *frankincense* ‘s potential as an antioxidant agent, providing fresh perspectives on its use for skin care products meant to lessen oxidative stress and the consequences of ageing, whereas earlier studies mainly concentrated on its usage for anti-inflammatory and antibacterial purposes. The Soxhlet extract’s superior efficacy in antibacterial and antioxidant tests further demonstrates how effective this technique is in removing bioactive substances, which makes it a perfect option for upcoming commercial uses. The study’s conclusions point to several important uses for *Boswellia sacra* extract: Antibacterial Skin Care goods because *frankincense* extract has antimicrobial properties against common skin pathogens, it could be used in goods like creams, lotions, and acne treatments. Anti-Ageing and Antioxidant Cosmetics, The Soxhlet extract’s strong antioxidant properties justify its application in anti-ageing products that shield the skin from oxidative damage brought on by free radicals. Products for Wound Healing and Anti-Inflammation: Modern preparations of *frankincense,* which include antimicrobial and anti-inflammatory qualities, can extend its historical usage as a wound-healing agent to treat cuts, abrasions, and mild skin infections.

Additionally, this study has several limitations that should be acknowledged. First, the antimicrobial evaluation relied solely on the agar well diffusion method, which is appropriate for preliminary screening but does not provide quantitative parameters such as the minimum inhibitory concentration (MIC) and minimum bactericidal/fungicidal concentration (MBC/MFC). These measurements are essential for determining the clinical relevance of the observed antibacterial effects and will be incorporated into future investigations. Second, antifungal testing was restricted to a single strain of *Candida albicans*, with no activity observed, limiting the generalizability of the antifungal findings. Further studies are needed to assess activity against a broader range of clinically significant fungal species, including dermatophytes and resistant strains. Third, the phytochemical screening produced positive results for all tested compound classes in both extracts, which raises concerns regarding the specificity of the qualitative methods used. Traditional colorimetric assays can be prone to cross-reactivity and false positives. Future work will incorporate advanced analytical techniques such as HPLC, LC-MS, and GC-MS to confirm and quantify individual bioactive compounds accurately. Fourth, extraction yield data were not recorded, preventing a quantitative comparison of the efficiency of Soxhlet and maceration techniques and leaving efficiency claims unsubstantiated. Fifth, inconsistencies in the antioxidant activity data (Table 6) were observed, with some concentrations not following the expected concentration–activity relationship. These deviations may result from experimental variability, limitations of the DPPH assay, or the complex composition of crude extracts, and they weaken the interpretation of antioxidant potency. Sixth, the evaluation of cream physical properties was largely subjective, relying only on visual and manual assessments without quantitative measurements such as viscosity, spreadability index, and texture analysis, which are crucial for scientific validation. Seventh, the cream formulation was not subjected to critical tests for product stability, including pH monitoring over time, accelerated stability under temperature cycling, preservative efficacy (challenge testing), and microbial safety assessments. These tests are vital to ensure product quality, safety, and shelf life. Finally, this study was limited to *in vitro* experiments, with no *in vivo* or clinical testing performed to validate the efficacy and safety of the *Boswellia sacra* extracts or the formulated cream in real-world applications. Future research should address these gaps by including quantitative yield determination, advanced phytochemical profiling, MIC/MBC analysis, expanded antifungal testing, detailed physicochemical and stability assessments of the cream, preservative efficacy and microbial safety evaluations, and clinical studies to enhance the reliability and translational relevance of the findings.

Future studies should focus on optimising the extraction process for large-scale production, as well as performing clinical trials to evaluate the efficacy and safety of *frankincense-based* products in humans. Furthermore, investigating synergistic combinations of *frankincense* extract with other plant-based substances may lead to more complete remedies for a wide range of dermatological issues.

## Conclusion

*Boswellia sacra* resin’s strong antibacterial and antioxidant qualities have been effectively shown in this work, underscoring its prospective uses in dermatological treatments as well as cosmetic formulations. Strong antibacterial activity was observed in the resin, especially against common skin infections *Pseudomonas aeruginosa* and *Staphylococcus aureus*. Its poor antifungal efficacy against *Candida albicans*, however, raises the possibility that bacterial infections rather than fungal ones would benefit more from its main application.

In comparison to the maceration approach, the Soxhlet extraction method demonstrated superior antibacterial capabilities and a higher antioxidant activity (84.66% RSA), making it the most effective method for extracting bioactive components. This study emphasises the importance of extraction techniques in maximising the bioactive potential of compounds derived from plants. Additionally, the resin’s viability for use in skincare products targeted at managing oxidative stress and skin infections is supported by the successful formulation of a topical cream containing *Boswellia sacra* extract that has favourable physical properties, such as smooth texture, excellent spreadability, and non-greasy consistency. Considering all factors, *Boswellia sacra* resin has demonstrated significant medicinal potential, particularly in the development of safe, natural alternatives to artificial antioxidants and antimicrobials. To evaluate the safety and effectiveness of *frankincense-based* formulations in human subjects, future research should concentrate on improving extraction techniques, looking into synergistic effects with other natural components, and carrying out clinical trials. These activities will further solidify *Boswellia sacra*’s position as a useful ingredient in the creation of safe and efficient skin care products.

## Data Availability

The original contributions presented in the study are included in the article/supplementary material, further inquiries can be directed to the corresponding author.
